# Synthesis of Silver Nanoparticles Using Azadirachta indica and Syzygium aromaticum Extract and Its Antibacterial Action Against Enterococcus faecalis: An In Vitro Study

**DOI:** 10.7759/cureus.65044

**Published:** 2024-07-21

**Authors:** Neena Chandran, Sindhu Ramesh, Rajeshkumar Shanmugam

**Affiliations:** 1 Conservative Dentistry and Endodontics, Saveetha Dental College and Hospitals, Saveetha Institute of Medical and Technical Sciences, Chennai, IND; 2 Nanobiomedicine Lab, Centre for Global Health Research, Saveetha Medical College and Hospitals, Saveetha Institute of Medical and Technical Sciences, Chennai, IND

**Keywords:** in vitro study, antibacterial activity, enterococcus faecalis, syzygium aromaticum, azadirachta indica, silver nanoparticles

## Abstract

Introduction

Nanotechnology is the study of manipulating matter at the atomic scale involving particles smaller than 100 nm. Silver nanoparticles (AgNPs) are gaining popularity across diverse sectors including medical, food, healthcare, consumer goods, and industrial fields due to their distinctive physical and chemical characteristics. The eco-friendly synthesis of AgNPs offers a straightforward, cost-effective, and environmentally benign method devoid of hazardous chemicals.

Methodology

Eighty milliliters (mL) of silver nitrate mixed with 20 mL of *Azadirachta indica* and *Syzygium aromaticum* plant extract underwent two days of magnetic stirring for AgNP synthesis. Characterization was done via ultraviolet-visible (UV-vis)-spectroscopy (300-700 nm), and antimicrobial properties, which were checked with *Enterococcus faecalis*, were assessed using the agar-well diffusion method.

Results

The change in color and peak observed in the UV-vis spectrum confirmed the successful synthesis of AgNPs. Both neem and clove extract-mediated synthesis of AgNPs exhibited antibacterial activity against *E. faecalis*. However, neem extract synthesized AgNPs displayed a larger inhibitory zone diameter and lower minimum inhibitory concentration (MIC) and minimum bactericidal concentration (MBC) values compared to those synthesized using clove extract.

Conclusion

Incorporating neem and clove extracts in AgNP synthesis offers a practical, eco-friendly, and cost-efficient method with notable efficacy. These AgNPs exhibit antibacterial activity against *E. faecalis*, suggesting their viability as potent antibacterial agents for addressing oral pathogens. Their sustainable synthesis underscores a promising avenue for developing effective antimicrobial solutions in oral healthcare.

## Introduction

Nanotechnology represents a cutting-edge realm of scientific inquiry focused on the fabrication, design, and manipulation of particles spanning 1 to 100 nm in size [1]. This field has catalyzed the emergence of low-dimensional materials endowed with distinctive attributes applicable across diverse industries such as textiles, medicine, electronics, and automation, positioning nanotechnology at the forefront of materials science [2].

Nanoparticles (NPs), deriving from a variety of metals and their oxides, offer a versatile toolkit for innovative applications. Among these metals, silver reigns supreme, prized for its adaptability, conductivity, versatility, and optical and electrical properties [3]. Silver nanoparticles (AgNPs) are extensively utilized across medical disciplines and are renowned for their potent antibacterial capabilities. Silver ions, with their ability to bind to proteins and phospholipids, disrupt bacterial function, underscoring silver's antimicrobial prowess [4].

The unique properties of AgNPs make them dynamic contenders in biological contexts, with remarkable attributes that include antifungal, antibacterial, antiviral, anti-infectious, wound-healing, and anti-inflammatory effects, earning silver the moniker of "dynamic" in the realm of biological applications [5]. Notably, AgNPs, when scaled to the nanosize regime (less than 100 nm), manifest distinct antibacterial properties compared to bulk silver. Their heightened toxicity against a spectrum of microbes and expanded surface area per individual NP enhance their efficacy in combating microbial toxicity [4,5].

Metal NPs can be synthesized through the reduction of metal salts in solution or atom aggregation, employing various techniques, including physical, chemical, and biological methods [6]. Among these, chemical reduction, due to its versatility, is the most widely utilized, particularly in the production of AgNPs [6]. Chemical reduction allows for the fabrication of AgNPs with tailored properties using diverse reduction precursors. However, the use of certain chemicals in NP synthesis can pose environmental and health risks, thereby necessitating the development of dependable, nontoxic, and environmentally friendly technologies for NP synthesis to expand their utility in biomedicine [7].

Green synthesis emerges as a promising alternative for NP synthesis, leveraging affordable and non-hazardous raw materials. This sustainable approach offers several advantages, including excellent therapeutic efficacy, low toxicity, tailored binding, and site-specific delivery [8]. Anastas and Warner's principles of "green chemistry" provide a framework for integrating environmentally benign practices into NP synthesis [7-9]. These principles emphasize reducing the reliance on hazardous chemicals and enhancing process effectiveness through methods such as preventing waste, designing safer chemicals and products, using renewable feedstocks, increasing energy efficiency, and minimizing the potential for accidents [10]. By adhering to these principles, researchers aim to mitigate the adverse effects associated with traditional chemical synthesis methods while advancing the development of novel nanomaterials for biomedical applications.

The plant-based biogenic approach stands out as a top method for large-scale production of NPs with precise size and morphology. Across diverse fields, including medicine, pharmaceuticals, clinical microbiology, and phyto-nanotechnology, researchers have explored plant systems as potential bio-factories for metal NPs [11]. Plant-based organic compounds, comprising alkaloids, phenolics, terpenoids, and coenzymes, have emerged as effective reducing and capping agents, facilitating environmentally friendly production of metal NPs.

Among the botanical candidates, neem, an evergreen tree known for its omnipotence, occupies a prominent position. Predominantly found in tropical regions of Africa and South Asia, especially on the Indian subcontinent, neem trees (scientifically named *Azadirachta indica* A. Juss.) belong to the Meliaceae family, which also includes mahogany trees [12]. *A. indica* flowers, leaves, seeds, and bark have been extensively utilized for their insecticidal, larvicidal, antimicrobial, antimalarial, antibacterial, antiviral, and anticancer properties [12]. The antibacterial prowess of *A. indica* can be primarily attributed to its key components, terpenoid and azadirachtin [13]. Notably, terpenoids and flavanones, the two significant phytochemicals in *A. indica*, serve dual roles as capping and reducing agents, crucial for stabilizing NPs [13]. By harnessing the inherent properties of *A. indica*-derived compounds, researchers aim to develop environmentally friendly strategies for producing metal NPs with tailored characteristics.

Clove, a highly commercialized species within the diverse genus *Syzygium*, stands out for its multifaceted applications. The *Syzygium *genus encompasses a plethora of woody species prized for their edible fruits, lumber, and medicinal properties, with clove (e.g., *Syzygium cumini*, *Syzygium aromaticum*, and *Syzygium jambos*) being among the most prominent [12,13]. Widely cultivated in China, Madagascar, Sri Lanka, and Indonesia, *S. aromaticum* is renowned for its fragrant flowers [13].

Historically, various *Syzygium* species have been utilized in traditional medicine to treat a wide array of ailments, contributing to their widespread recognition. The essential oil derived from *S. aromaticum* is traditionally used to heal burns, wounds, dental pain, and tooth infections [14]. Moreover, its antibacterial properties render it a popular choice for food preservation. Beyond antibacterial efficacy, reports suggest additional therapeutic benefits such as antioxidant, analgesic, anesthetic, anti-inflammatory, and insecticidal actions [15]. Moreover, its antibacterial properties render it a popular choice for food preservation, effectively inhibiting the growth of foodborne pathogens and spoilage bacteria. Studies have demonstrated its ability to disrupt bacterial cell walls, interfere with enzyme activities, and prevent biofilm formation, thereby extending the shelf life of perishable goods. Beyond antibacterial efficacy, reports suggest additional therapeutic benefits such as antioxidant, analgesic, anesthetic, anti-inflammatory, and insecticidal actions. This multifaceted utility underscores its potential in various applications, from healthcare to agriculture [13-15].

The exploration of diverse pharmacological attributes of *S. aromaticum* underscores its potential as a valuable resource in modern healthcare and culinary applications. Screening medicinal plants for bioactive compounds holds promise for the development of novel antimicrobial medications that are not only more affordable but also safer and more effective. The plant parts utilized in this study, namely, *A. indica* and *S. aromaticum*, are easily accessible in both rural and urban areas of India and are widely accepted by the population. This study aimed to investigate the antibacterial potential of Ag NPs synthesized using *A. indica* and *S. aromaticum* against *Enterococcus faecalis*, given their recognized antibacterial properties. Biocompatible AgNPs were synthesized using a biological method, wherein plant extracts served as capping and stabilizing agents to convert silver ions into NPs. To date, no comparative study has been conducted to evaluate the antimicrobial activity of AgNPs synthesized from natural extracts of *A. indica* and *S. aromaticum* against *E. faecalis*.

Therefore, this study fills a critical gap in the literature and provides valuable insights into the antibacterial efficacy of AgNPs produced through a biological approach against *E. faecalis*.

## Materials and methods

The aim of this study was to compare the antimicrobial efficiency of NPs derived from plant extracts against E. faecalis in an in vitro setting. All chemicals utilized in the research were purchased from Sigma-Aldrich (St. Louis, MO, USA) and were of analytical grade, ensuring high quality and reliability of results.

Synthesis of *A. indica* AgNPs (AiNPs) and *S. aromaticum* AgNPs (SaNPs)

To synthesize AgNPs, aqueous extracts of *A. indica* leaves and *S. aromaticum* buds were utilized. The leaves and buds were collected and thoroughly washed with distilled water. Subsequently, they were air-dried at room temperature for three days to remove any contaminants. The dried plant materials were then processed to obtain aqueous extracts, which served as reducing and stabilizing agents in the synthesis of AgNPs. This method ensured the utilization of natural bioactive compounds present in *A. indica* and *S. aromaticum*, contributing to the eco-friendly and biocompatible nature of the synthesized NPs [14].

Preparation of plant extract [15]

The dried leaves of *A. indica* and spices like *S. aromaticum* were processed into a coarse powder using a blender. Subsequently, 1 g of this powder was combined with 100 mL of distilled water and heated to 55°C for 15 minutes using a heating mantle. Following this, the mixture was allowed to cool to room temperature and then filtered using Whatman No. 1 filter paper. This method facilitated the extraction of bioactive compounds from the plant materials, which were essential for the synthesis of AgNPs.

Synthesis of AgNPs

The silver nitrate (AgNO3) salt (99.9%) used in this study was sourced from Sigma-Aldrich (209139-25G). A 0.1 M stock solution was prepared by dissolving 10 mM of AgNO3 in 80 mL of distilled water and stored in the dark to prevent light-induced reactions. Subsequently, 80 mL of the AgNO3 solution was combined with 20 mL of the plant extract solution and placed on a magnetic stirrer for two days. To ensure thorough mixing, the solution was transferred to an orbital shaker at 65 rpm and later to a magnetic stirrer at 450 rpm.

During the two-day synthesis period, the color change of the solution was monitored at hourly intervals, serving as an indicator of AgNP formation. Additionally, ultraviolet (UV) spectroscopy was employed to confirm the synthesis of AgNPs by analyzing the characteristic absorption peaks. Following synthesis, the solution underwent centrifugation at 10,000 rpm for 10 minutes to separate the NPs from the solution. Dilutions were then prepared by mixing 25, 50, and 100 µL of the *A. indica* and *S. aromaticum* solution with sterile water and stored in sealed containers for further analysis. This meticulous process ensured the controlled synthesis and characterization of AgNPs derived from plant extracts.

Characterization of AgNPs

The characterization of the eco-friendly synthesized AiNPs and SaNPs was conducted using ultraviolet-visible (UV-vis) spectroscopy. Confirmation of AgNP formation was achieved by analyzing spectral data obtained from UV-vis analysis. For this analysis, a 3 mL solution was placed in a cuvette and scanned in a double-beam UV-vis spectrophotometer (ELICO SL 210 UV-vis spectrophotometer) across a wavelength range of 300-700 nm. The obtained data were meticulously recorded and graphically analyzed to identify characteristic absorption peaks indicative of AgNP formation. This method allowed for the quantitative assessment of NP synthesis and provided valuable insights into the optical properties of the synthesized AiNPs and SaNPs. The UV-vis spectroscopy analysis served as a fundamental step in confirming the successful synthesis of AgNPs using eco-friendly methods derived from *A. indica* and *S. aromaticum* plant extracts.

Antimicrobial assays of biosynthesized AgNP

The organism utilized in this study was *E. faecalis*, a gram-positive, facultative anaerobe commonly found in the human gastrointestinal tract, obtained from the culture collections of the Nanobiomedicine Laboratory, Saveetha Dental College, Chennai. The laboratory investigation spanned a duration of eight weeks. To assess the antibacterial effects of different concentrations of plant extracts, various methods, including minimum inhibitory concentration (MIC) determination in test tubes, minimum bactericidal concentration (MBC) determination in plate culture medium, and measurement of growth inhibition zone diameter, were employed.

Antimicrobial susceptibility test

The antimicrobial activity testing was conducted using the Kirby-Bauer susceptibility method, a well-established technique in microbiology [16]. Mueller-Hinton agar (MHA) was selected as the medium for this activity due to its standardized composition and consistent results in determining the zone of inhibition (ZOI), a key indicator of antimicrobial activity. The agar was meticulously sterilized at 121°C for 15 minutes to ensure the absence of any contaminants that could interfere with the test results. Once sterilized, the agar was poured into sterile plates and allowed to solidify, providing a stable surface for bacterial growth and diffusion of antimicrobial agents. Using a 9 mm sterile polystyrene tip, wells were carefully cut into the agar surface, creating spaces for the application of test substances. Test organisms were then swabbed onto the agar surface, evenly distributing the bacterial culture. Subsequently, different concentrations of NPs (25, 50, and 100 µL) were loaded into the designated wells. Additionally, a positive control containing sodium hypochlorite and a negative control containing saline were included to validate the assay results [15].

Following inoculation, the plates were incubated for 24 hours at 37°C, optimal conditions for bacterial growth. During this incubation period, the antimicrobial agents diffused through the agar, exerting their inhibitory effects on bacterial growth. After incubation, the plates were carefully examined, and the ZOI surrounding each well was measured using standardized techniques. The diameter of the ZOI provided a quantitative measure of the antimicrobial activity of the tested substances against the target organism, *E. faecalis*. Overall, this protocol, based on the Kirby-Bauer disk diffusion susceptibility test, facilitated the comprehensive assessment of the antimicrobial efficacy of NPs derived from plant extracts. By employing standardized methods and controls, the study ensured reliable and reproducible results, essential for the accurate evaluation of antimicrobial agents.

MIC

To determine the MIC of AgNPs against *E. faecalis*, Mueller Hinton broth was prepared and sterilized to provide a standardized growth medium. Subsequently, 6 mL of broth was dispensed into three test tubes, followed by the addition of an overnight bacterial suspension, with concentrations ranging from 5 × 10^5^ CFU/mL. Various concentrations of AgNPs (25, 50, and 100 µL) were then added to the test tubes, along with positive control (sodium hypochlorite) and negative control (saline) for comparison. The test tubes were then incubated at 37°C for varying time intervals (one, two, three, four, and five hours) to assess the inhibitory effect of AgNPs on bacterial growth.

To quantify the inhibitory effect, the percentage of dead cells was calculated at regular intervals by measuring the absorbance at a wavelength of 600 nm. This allowed for the determination of the concentration of AgNPs required to inhibit bacterial growth, represented by the MIC. By systematically evaluating the impact of AgNPs on *E. faecalis* growth over time, this approach provided valuable insights into the antimicrobial efficacy of AgNPs and their potential as alternative therapeutic agents against bacterial pathogens.

MBC by agar dilution method

The MBC, representing the lowest concentration of antibacterial agents required to completely eradicate bacterial growth, was determined using the MBC test. MHA plates were prepared by autoclaving and pouring the agar onto sterile petri plates, allowing it to solidify. For the MBC test, the MHA plates were swabbed with aerobic bacterial suspension and inoculated with three different concentrations of neem and clove, along with standard and positive controls. The plates were then incubated for 24 hours to facilitate bacterial growth.

Following the incubation period, the plates were carefully observed, and the number of colonies present on each plate was counted. The lowest concentration of *A. indica* and *S. aromaticum* NPs that did not exhibit any visible bacterial growth on the MHA plate was determined as the MBC value. This method allowed for the precise determination of the concentration of Ag NPs required to exert bactericidal effects against the target pathogen, *E. faecalis*. By identifying the MBC value, the effectiveness of *A. indica* and *S. aromaticum* NPs as antimicrobial agents could be quantitatively assessed, providing valuable information for their potential use in combating bacterial infections.

Statistical analysis

Sodium hypochlorite served as the positive control, while saline was used as the negative control to ensure the validity of the experimental results. The obtained data were analyzed using SPSS (IBM SPSS Statistics for Windows, IBM Corp., Version 24, Armonk, NY) to ascertain the significance of the antibacterial effects observed. This rigorous analytical approach facilitated the comparison of the efficacy of plant extract concentrations against *E. faecalis*, providing valuable insights into their potential as alternative antimicrobial agents [16].

## Results

Characterization of AgNPs

The study elucidated that the introduction of plant extract into the AgNO3 solution induced a discernible change in color, attributed to the localized surface plasmon resonance (SPR) phenomenon exhibited by the formed AgNPs. Analysis of the UV-vis spectrum revealed a prominent SPR peak, indicative of the production of polydisperse NPs. This phenomenon arises from the presence of free electrons in the metal NPs, leading to the absorption of light waves at specific wavelengths. The collective oscillation of electrons within the metal NPs, in conjunction with incident light, results in the plasmon resonance peak absorption band observed in the UV-vis spectrum.

Similar observations were reported in previous studies, where the synthesis of AgNPs using extracts from *A. indica* and *S. aromaticum* also led to a noticeable color change in the reaction mixture. These findings corroborate the efficacy of plant extracts as reducing and stabilizing agents in the eco-friendly synthesis of AgNPs and underscore the widespread applicability of this approach across different botanical sources.

**Figure 1:**
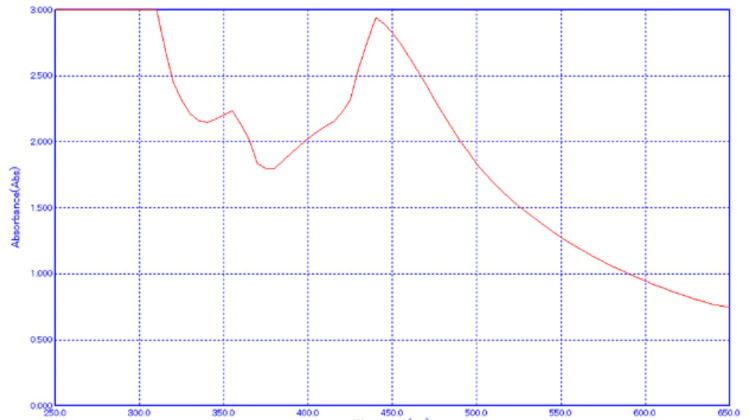
UV-vis spectroscopy of Azadirachta indica AgNPs UV-vis: ultraviolet-visible; AgNPs: silver nanoparticles

**Figure 2:**
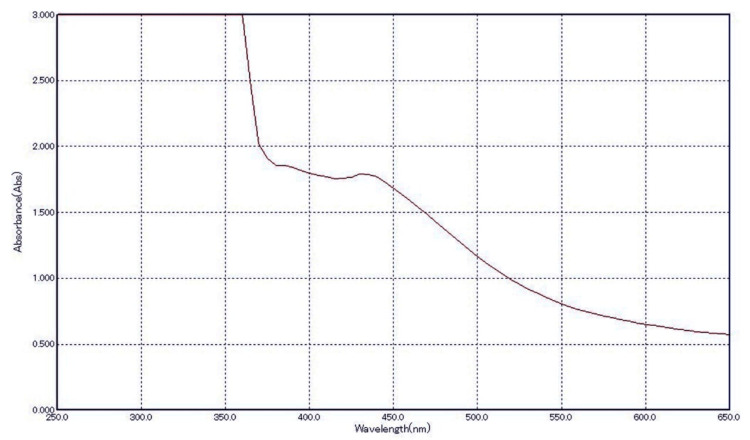
UV-vis spectroscopy of Syzygium aromaticum AgNPs UV-vis: ultraviolet-visible; AgNPs: silver nanoparticles

Figure 1 and Figure 2 depict the absorption peak of the AgNPs synthesized using AgNO3 and extracts of *A. indica* and *S. aromaticum*. The absorption peak for *A. indica* AgNPs is observed at 440 nm, while for *S. aromaticum* AgNPs, it ranges from 425 to 480 nm. These values fall within the typical range of 350 to 600 nm detected for colloidal AgNPs in previous studies. The differences in the UV spectra of the AgNPs can be attributed to their distinct physical properties, such as size and shape, which significantly influence their SPR characteristics. These variations underscore the importance of considering the specific synthesis methods and precursor materials employed in NP production when analyzing their optical properties.

Antibacterial assay

**Figure 3:**
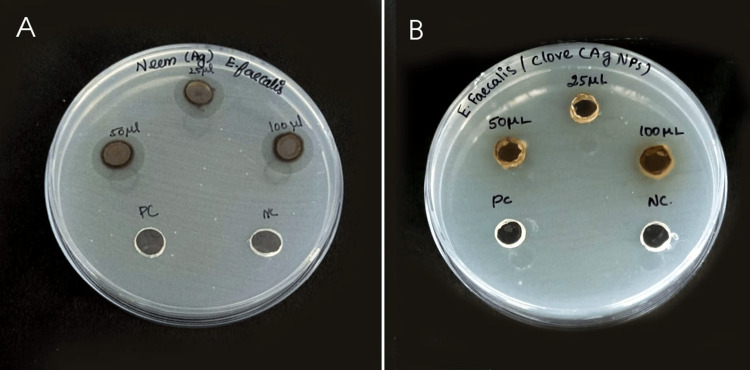
Disk diffusion assay showing the visible clear zone produced by AgNP synthesis using plant extract against Enterococcus faecalis. (A) Azadirachta indica AgNPs (B) Syzygium aromaticum AgNPs AgNPs: silver nanoparticles

An assay was conducted to assess the antibacterial efficacy of AgNPs derived from *A. indica* and *S. aromaticum* against *E. faecalis* using the disk diffusion method. The formation of a clear zone around the AgNPs disk indicated their possession of antibacterial properties capable of inhibiting the growth of Gram-positive pathogens. Figure 3 presents an image depicting the visible clear zone generated by the biosynthesized AgNPs when tested against *E. faecalis*. This observation underscores the potential of AgNPs derived from natural sources such as *A. indica* and *S. aromaticum* as effective antimicrobial agents against bacterial pathogens, highlighting their promising role in combating infectious diseases. Figure 3 depicts the ZOI, with maximum ZOI at 100 µL.

**Figure 4:**
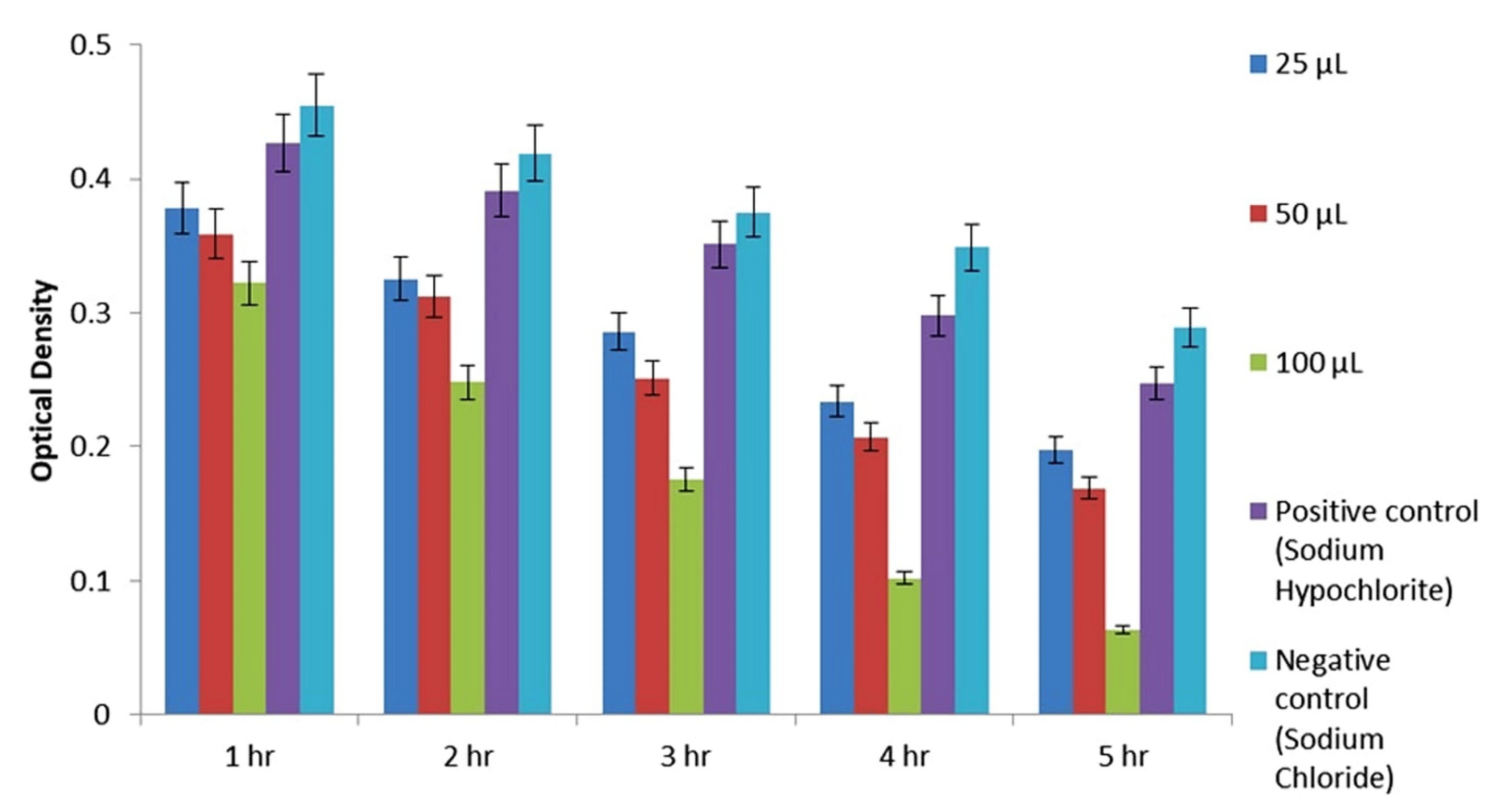
MIC of Azadirachta indica AgNPs AgNPs: silver nanoparticles; MIC: minimum inhibitory concentration *p value < 0.01

**Figure 5:**
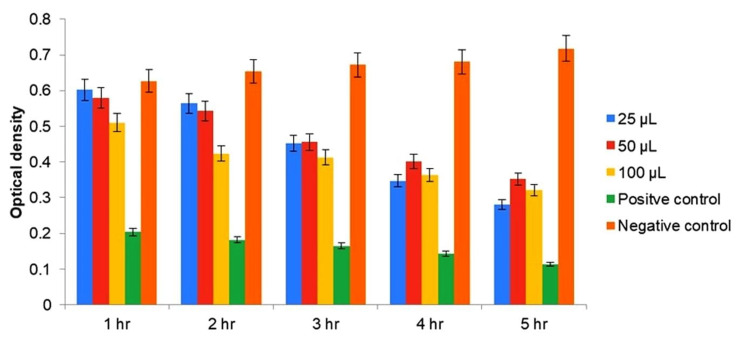
MIC of Syzygium aromaticum AgNPs AgNPs: silver nanoparticles; MIC: minimum inhibitory concentration *p value < 0.01

The results of the disk diffusion test for AgNPs are summarized in Table 1. AgNPs derived from *A. indica* exhibited a larger ZOI of 16 mm at a concentration of 100 μL. However, it is important to note that the disk diffusion test serves as a preliminary study to screen the antibacterial activity of an antimicrobial agent. Further evaluation of the antibacterial activity of AgNPs was conducted using the MIC value, which represents the smallest amount of an antibacterial agent required to halt bacterial growth through serial dilution. Figure 4 and Figure 5 depict the MIC values of both *A. indica* AgNPs and *S. aromaticum* AgNPs against *E. faecalis* at various time intervals.

**Table 1:** Zone of inhibition

Samples	25 µL	50 µL	100 µL	Positive control	Negative control
Neem (Ag)	13	14	16	9	1
Clove (Ag)	9	10	12	9	1

The MBC represents the minimum amount of antibacterial agent required to completely eradicate bacteria, leading to no visible growth on the agar plate. The colony-forming unit (CFU) counts presented in Table 2 suggest that the inclusion of AgNPs derived from *A. indica* and *S. aromaticum* extracts resulted in varying degrees of inhibition of bacterial growth. Notably, the CFU count for *A. indica* AgNPs at a concentration of 100 μL was substantially lower compared to other samples, indicating the efficacy of the antimicrobial agent at this concentration in inhibiting bacterial growth. Conversely, the negative control exhibited the highest CFU count, as expected, since no antimicrobial agent was present in that sample. These findings underscore the effectiveness of AgNPs derived from natural sources, such as *A. indica* and *S. aromaticum*, in inhibiting bacterial growth and highlight their potential as antimicrobial agents against bacterial pathogens.

**Table 2:** MBC of A. indica AgNPs and S. aromaticum AgNPs AgNPs: silver nanoparticles; *A. indica*: *Azadirachta indica*; CFU: colony-forming unit; MBC: minimum bactericidal concentration; *S. aromaticum*: *Syzygium aromaticum*

AgNPs	25 μL	50 μL	100 μL	Positive control	Negative control
*A. indica*	35 × 102 CFU/mL	35 × 102 CFU/mL	17 × 103 CFU/mL	287 × 104 CFU/mL	65 × 104 CFU/mL
*S. aromaticum*	Enormous growth	Enormous growth	Enormous growth	Enormous growth	Enormous growth

The synthesis of AgNPs using *A. indica* and *S. aromaticum* extracts resulted in varying levels of bacterial growth inhibition, as indicated in Table 2. Notably, *A. indica* AgNPs at a concentration of 100 μL exhibited a significantly lower CFU count compared to other samples, highlighting the effectiveness of the antimicrobial agent in halting bacterial growth at this concentration. Conversely, the negative control sample displayed the highest CFU count, as expected, due to the absence of any antimicrobial agent. In contrast, *S. aromaticum* AgNPs at different concentrations showed considerable bacterial growth, suggesting poor antibacterial efficacy. These findings underscore the differential antimicrobial activity of AgNPs derived from different botanical sources and emphasize the need for further investigation to optimize their efficacy against bacterial pathogens.

Visual observation

**Figure 6:**
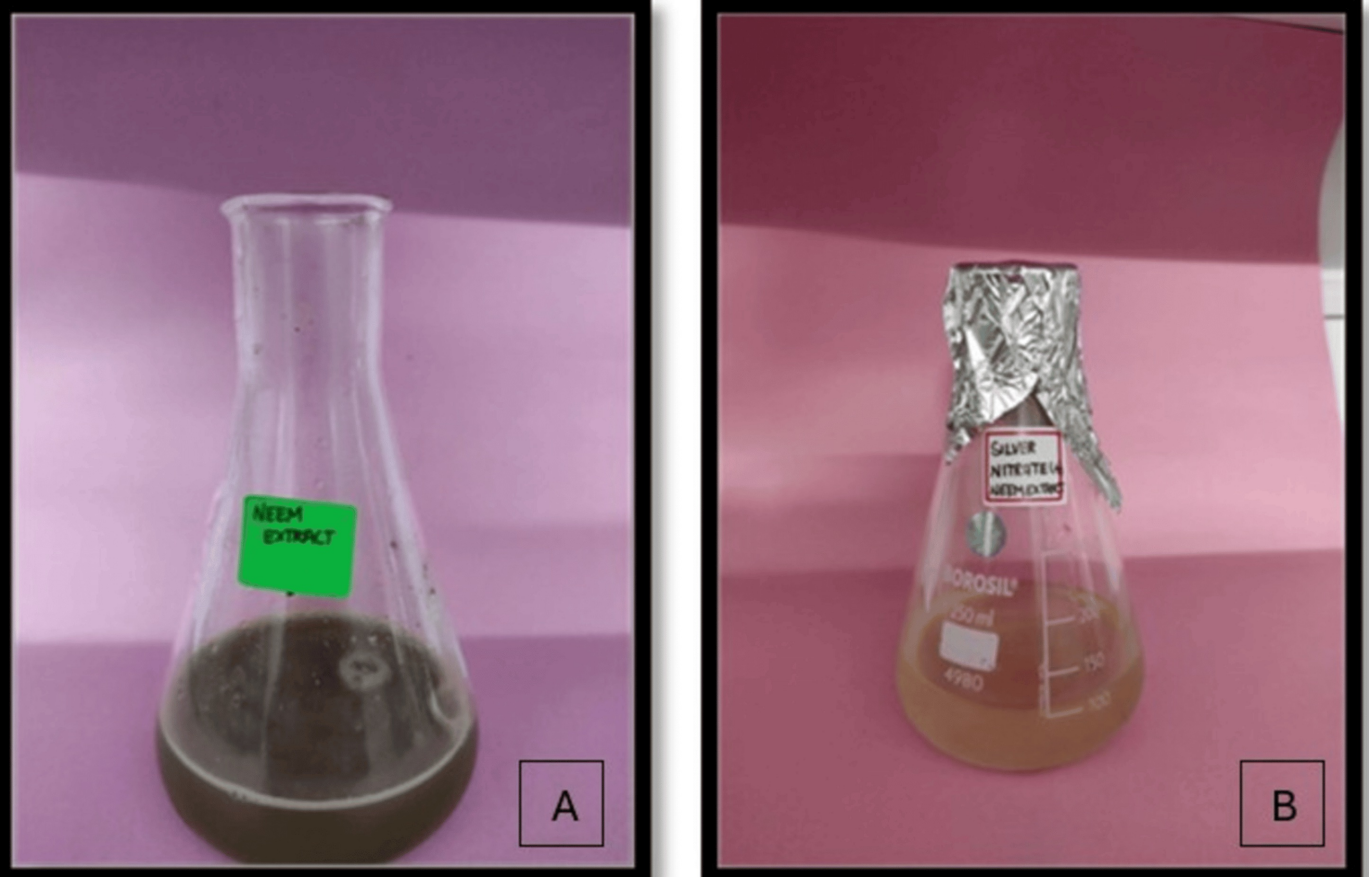
Synthesis of silver nanoparticles from Azadirachta indica extract indicated by color change from (A) brown to (B) pale yellow

**Figure 7:**
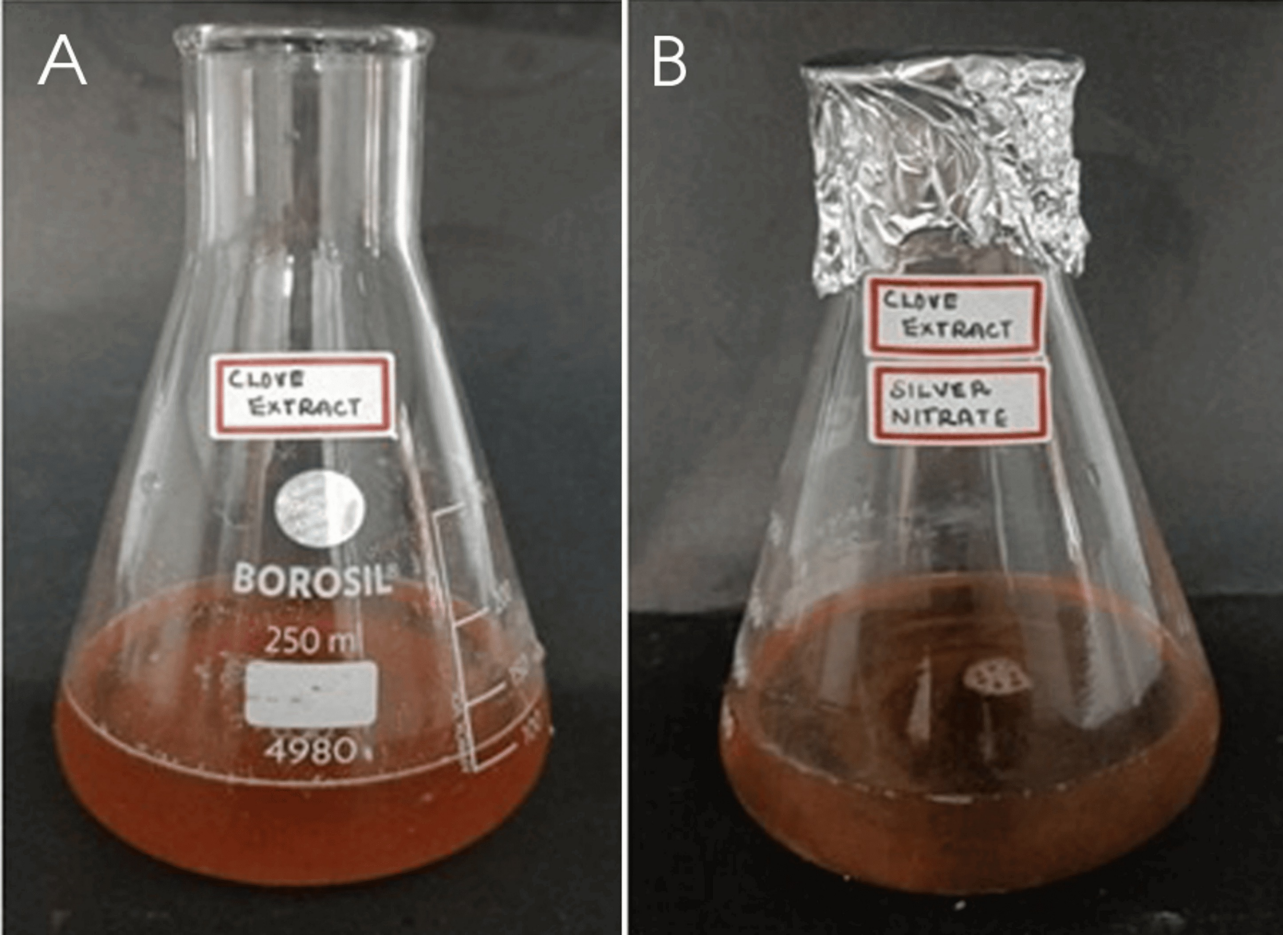
Synthesis of silver nanoparticles using Syzygium aromaticum extract: visual confirmation through color change

The synthesis of AgNPs using extracts from *A. indica* (neem) and *S. aromaticum* (clove) was visually confirmed through distinct color changes, as depicted in Figure 6 and Figure 7. In the case of *A. indica*, the reaction mixture changed from brown to pale yellow, indicating the successful formation of AgNPs. Similarly, the synthesis using *S. aromaticum* extract was confirmed by an observable color shift, providing visual evidence of NP formation. These color changes are characteristic of the reduction process of silver ions to AgNPs, showcasing the effectiveness of these plant extracts in facilitating NP synthesis.

## Discussion

In contrast to chemical and physical methods, which can lead to the adsorption of harmful substances onto the NP surface, rendering them unsuitable for medical applications, biological approaches offer the advantage of generating more biocompatible NPs [16,17]. Plants and plant-derived materials contain a plethora of metabolites with redox potential that serve as reducing agents during the biogenic synthesis of NPs. Moreover, plant-based synthesis yields highly stable NPs produced at a faster rate than microbial synthesis [18].

This accelerated synthesis rate may be attributed to the abundance of biologically active compounds present in plants, including flavones, ketones, aldehydes, amides, carboxylic acids, proteins, DNA, and enzymes, which act as bio-reducing agents [19]. These compounds facilitate the conversion of Ag ions to AgNPs [20]. Consequently, the utilization of plant-based synthesis not only ensures the production of biocompatible NPs but also offers advantages, such as enhanced stability and efficiency in NP synthesis, making it a promising approach for various biomedical applications [21].

The study investigated the efficacy of AgNPs derived from *A. indica* and *S. aromaticum* extracts in inhibiting the growth of *E. faecalis*, a prevalent species implicated in various oral infections such as marginal periodontitis, infected root canals, peri radicular abscesses, and failed endodontic therapy. Both *A. indica* and *S. aromaticum* AgNPs exhibited antibacterial activity against *E. faecalis*, with identical MIC values of 100 µL [22]. Specifically, the aqueous extract of *A. indica* demonstrated the highest mean ZOI, measuring 16 mm at 100 µL, whereas *S. aromaticum* extract displayed a slightly lower mean ZOI of 12 mm at the same concentration. These findings underscore the potential of AgNPs synthesized from natural sources such as *A. indica* and *S. aromaticum* extracts as effective antimicrobial agents against *E. faecalis*, highlighting their promising role in the treatment and management of oral infections associated with this pathogenic microorganism.

The precise mechanism underlying the antibacterial action of AgNPs remains elusive despite their widespread use. However, it has been observed that AgNPs, when bound to the bacterial cell membrane surface, can induce instability in membrane permeability and respiratory activity, leading to antibacterial effects [23,24]. Additionally, the antibacterial properties of *A. indica* and *S. aromaticum* extracts have been extensively investigated by researchers. For instance, Regmi et al. (2021) conducted a study on the antibacterial activity of AgNPs synthesized using *A. indica* leaf extract against *E. faecalis*, reporting a ZOI of 12 mm, which underscores the effectiveness of AgNPs in combating bacteria [25]. Moreover, Nwali et al. (2018) found that the leaves of *A. indica* contained higher concentrations of alkaloids, flavonoids, terpenoids, and saponins compared to the stem bark and root, suggesting their potential role in imparting antibacterial properties to the plant extract [26].

These findings contribute to the understanding of the potential mechanisms underlying the antibacterial activity of AgNPs and highlight the importance of exploring natural sources, such as neem and clove extracts, for their antimicrobial properties, which could have significant implications for the development of novel antibacterial agents.

Despite the promising antibacterial properties of AgNPs synthesized from *A. indica* and *S. aromaticum* extracts, this study has certain limitations. First, the exact biochemical pathways and interactions through which these plant extracts facilitate NP synthesis remain incompletely understood, necessitating further research to elucidate these mechanisms fully. Second, while the study demonstrated efficacy against *E. faecalis*, a comprehensive evaluation across a broader spectrum of pathogenic microorganisms is essential to confirm the generalizability of these findings. Additionally, the in vitro conditions under which these NPs were tested may not perfectly replicate the complex biological environment in vivo, raising questions about their practical applicability and effectiveness in clinical settings. Furthermore, the potential cytotoxicity and biocompatibility of these NPs must be thoroughly assessed to ensure safety for medical applications. Lastly, variations in the concentration and composition of bioactive compounds in plant extracts due to geographical and environmental factors could lead to inconsistencies in NP synthesis and efficacy, highlighting the need for standardized extraction and synthesis protocols.

## Conclusions

In conclusion, this study has successfully demonstrated the efficient production of antibacterial AgNPs using plant extracts derived from *A. indica* and *S. aromaticum*, along with their effectiveness against *E. faecalis*, a common oral pathogenic bacterium. The synthesis of AgNPs using *A. indica* and *S. aromaticum* extracts exhibited potent antibacterial activity against *E. faecalis*. Notably, *A. indica* extract-synthesized AgNPs demonstrated superior efficacy compared to clove extract-synthesized AgNPs, as clove-based NPs did not have significant antibacterial activity. This study underscores the potential application of AgNPs synthesized from natural plant extracts as effective antibacterial agents against *E. faecalis*.

Moreover, the utilization of plant extracts for AgNP synthesis presents an eco-friendly, cost-effective, and efficient approach that may offer a safer and more sustainable alternative to conventional methods of AgNP synthesis. However, further investigations are warranted to evaluate the efficacy and safety of these AgNPs in vivo and to explore their potential clinical applications. Such endeavors will contribute to advancing our understanding of the therapeutic potential of plant-extract-synthesized AgNPs and their role in combating bacterial infections, particularly in the field of oral healthcare.

## Figures and Tables

**Figure 8:**
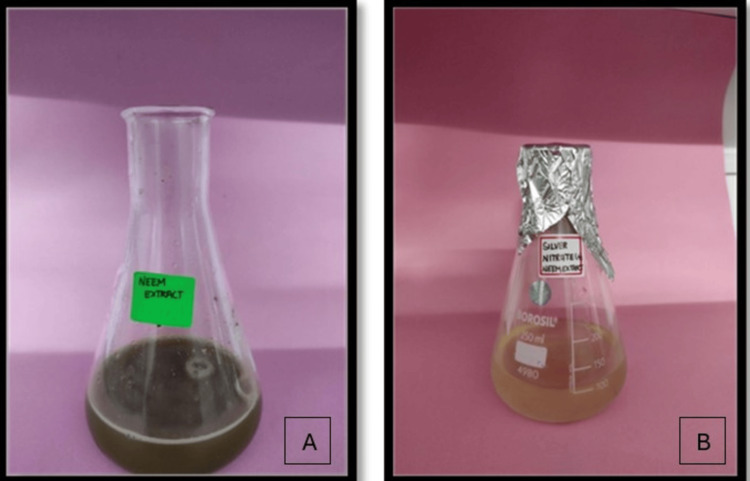
Synthesis of silver nanoparticles from Azadirachta indica extract indicated by colour change from A. Brown to B. Pale Yellow

**Figure 9:**
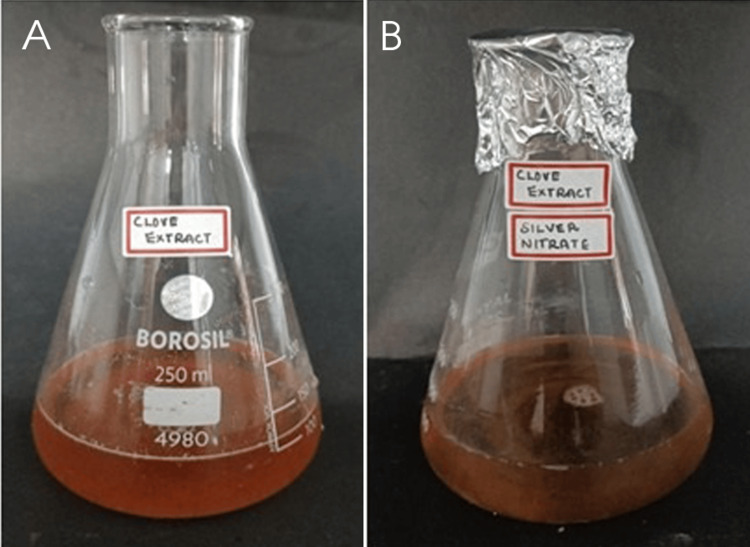
Synthesis of Silver Nanoparticles Using Syzygium Aromaticum Extract: Visual Confirmation through Colour Change
